# Supplementary material for: Self-reported menses physiology is positively modulated by a well-formulated, energy-controlled ketogenic diet vs. low fat diet in women of reproductive age with overweight/obesity
Source: PLoS One. 2024 Aug 16;19(8):e0293670. doi: 10.1371/journal.pone.0293670 (PMC11329152; doi:10.1371/journal.pone.0293670)
Supplement: S1 File — (PDF) [file pone.0293670.s001.pdf]

IRB Protocol

Title: Strategies to Augment Ketosis

PI: Jeff S. Volek, PhD, RD
Professor, Department of Human Sciences
The Ohio State University
305 Annie & John Glenn Ave
Columbus, OH 43210
Office: 614-688-1701 | volek.1@osu.edu

Co-Investigators: Orlando P. Simonetti, PhD
John W. Wolfe Professor in Cardiovascular Research
Professor, Internal Medicine and Radiology
Division of Cardiovascular Medicine
The Ohio State University
Office: 614-293-0739 | orlando.simonetti@osumc.edu

William J. Kraemer, PhD
Professor, Department of Human Sciences
The Ohio State University
Office: 614-688-2354 | kraemer.44@osu.edu

SUMMARY AND BACKGROUND

Because of the historical negative connotation of ketones (ketones were first discovered in the urine of uncontrolled type-1 diabetics in keto-acidosis) and the mainstream bias against ketogenic diets, nutritional ketosis has been understudied by scientists and under-utilized by clinicians. That is changing rapidly as new functions and applications of ketones come to light. Hepatic production of the 4 carbon ketones beta-hydroxybutyrate (BOHB) and acetoacetate is increased when insulin levels are low enough to result in accelerated lipolysis (breakdown of adipose tissue triglycerides) and increased release of fatty acids into the circulation. Ketones are synthesized from the partial breakdown of fatty acids in the liver, a process called '*ketogenesis*'. Release of fatty acid-derived ketones into the circulation results is a metabolic state of '*ketosis*'.

The liver produces ketones all the time, but the rate of ketogenesis and level of ketosis depends on dietary carbohydrate and protein intake. When enough carbohydrate and protein is consumed to elevate the hormone insulin to levels that inhibit fat breakdown (and make glucose the predominant fuel), then ketogenesis operates at a low level translating into blood ketone concentrations around 0.1 mmol/L. Given the heavy emphasis on carbohydrate, most people rarely experience ketones higher than 0.3 mmol/L.

When carb and protein intake contribute less than ~20% of total energy expenditure (i.e., a ketogenic diet that contains ~40 grams carbohydrate and ~90 grams protein), insulin levels are depressed, glucose is in short supply, and the ketogenic pathway accelerates resulting in a natural state of '*nutritional ketosis*' characterized by ketone concentrations between 0.5 and 2 mmol/L for most people consuming a well formulated ketogenic diet including a variety of non-starchy vegetables. Levels fluctuate throughout the day depending on exercise and food intake, and may transiently go as high as 5 mmol/L.

When maintained for several consecutive weeks, nutritional ketosis fundamentally changes the way cells work. This includes switching the fuel they use, as well as awakening genes that are silenced by high-carb diets. Over time the body refines itself to run on fat and ketones ultimately manifesting in a keto-adapted phenotype characterized by two-fold higher rates of whole body fatty acid oxidation, while glycolysis, insulin concentrations and cell signaling, constitutive inflammation and oxidative stress are all decreased. Keto-adaptation has therapeutic effects; most notable reversal of metabolic syndrome and type-2 diabetes, but many other clinical conditions may be amendable to nutritional ketosis.

It is now possible to rapidly elevate blood ketones by ingesting BOHB in supplement form. The resultant '*acute nutritional ketosis*' does not require a ketogenic diet or any carb and protein restriction. Supplemental ketones are absorbed from the gut into the circulation and thus does not involve increased hepatic ketogenesis. The level of ketosis varies depending on the form and the dose, but concentrations can acutely elevate into the range of nutritional ketosis. The effect is short-lived lasting a few hours, thereby requiring repeated dosing to achieve sustained ketosis.

It remains unknown how combining a ketogenic diet with ketone supplements affects ketosis, weight loss, and other features of the keto-adapted phenotype. This project will shed light on whether adding exogenous ketones to a ketogenic diet affects initial adaptations over a 6 wk period. Primary outcomes will include ketosis, weight loss, body composition, and a host of other metabolic and cognitive outcomes. All patients are expected to lose weight since we will be feeding them a calorically-controlled ketogenic diet and experience improvements in health with minimal risk.

Summary Existing Knowledge

Work on human starvation over 4 decades ago discovered a series of elegant metabolic adaptations; notably acceleration of hepatic ketone production to displace the majority of brain glucose utilization, conferring a remarkable protection from hypoglycemia and other neuro-protective effects (1). The plasticity to decrease glucose dependency during starvation ketosis had a critical role during human evolution, but today these pathways are silenced by the ubiquitous presence of and emphasis on carbohydrate as a dietary staple. Interest in ketone physiology has surged in the last decade contributing to a critical mass of discoveries that link the primary circulating ketone beta-hydroxybutyrate (BOHB) and the process of keto-adaptation to broad-spectrum health benefits (2-6).

Similar to starvation ketosis, diets restricted in carbohydrate accelerate ketogenesis resulting in circulating ketones between 0.5 and 4.0 mmol/L. Recent evidence indicates that BOHB is a potent cellular signaling molecule that positively affects gene expression, protection from oxidative stress, insulin resistance, and longevity (3-8). A chronic state of nutritional ketosis manifests in a keto-adapted phenotype characterized by two-fold higher rates of whole body fatty acid oxidation, while glycolysis, insulin concentrations, constitutive inflammation and oxidative stress are all decreased (3,8-9). Keto-adaptation reverses the sequela associated with the insulin resistant phenotype including metabolic syndrome and type-2 diabetes (5,8,10).

RESEARCH OBJECTIVES

1. The primary objective is to examine if supplementing with exogenous β-hydroxybutyrate (BOHB) as part of a hypocaloric ketogenic diet designed to achieve ketosis and weight loss has any impact on indices of keto-adaptation in obese subjects.

2. A secondary objective is to determine the time-course of early phases of keto-adaptation with and without exogenous ketone supplements.

Key outcome variables will include 4 major thrusts:

1) MARKERS OF KETOSIS/RENAL ADAPTATIONS

a. Daily measures of fasting BOHB will be determined from capillary blood. Participants will be provided with a glucometer (Precision Xtra, Abbott Nutrition) and ketone test strips to check the concentration of BOHB from a finger stick each morning. This will give us a daily fasting measurement of ketosis over the 6-wk feeding period.
b. Fasting venous blood will be collected bi-weekly for analysis of serum total ketones and BOHB (other measures will be analyzed as well)
c. Diurnal ketone patterns will be determined bi-weekly (the same day we collect fasting blood). Participants will test capillary BOHB and glucose hourly during awake hours (12-hr total) to provide a diurnal ketosis pattern over the course of the day. During the same period, participants will collect their urine for measurement of total ketones and BOHB.
d. We will also measure several renal markers in the 24 hr urine including creatinine, nitrogen, uric acid, electrolytes, and other measures of renal function.

2) ENERGY BALANCE AND BODY COMPOSITION

a) Body mass will be measured weekly on a calibrated digital scale in our laboratory.
b) Body composition will be determined bi-weekly by dual-energy x-ray absorptiometry (iDXA, Lunar Corporation, Madison, WI). One whole body scan will quantify lean and fat mass using encore software and visceral fat quantification will be calculated using the new CoreScan software.
c) On the same day we assess 12-hr diurnal ketones, we will have subjects extend their urine collection to 24-hr to assess daily nitrogen loss. As a measure of anabolism, we will calculate nitrogen balance from estimates of nitrogen intake and loss.
d) Resting metabolic rate (RMR) and substrate oxidation will be determined bi-weekly by indirect calorimetry. Participants will be asked to abstain from food and beverages for a minimum of 12-hr, alcohol and caffeine for 24-hr and

physical activity for 48 hr before testing. The test will be conducted using a metabolic cart (TrueOne 2400, Parvomedics Inc. Sandy, UT). After a 30 min rest period, continuous measures of CO_2 and O_2 will be averaged and recorded every 30 sec during 30 min of testing and used to calculate RMR and substrate oxidation.

e) Measures of visceral adipose tissue and organ fat will be determined from magnetic resonance imaging (MRI). Visceral adipose tissue volume will be determined from MRI scans acquired using a Siemens 3T MAGNETOM Prisma at baseline and wk-6. Visceral fat will be determined from multiple-slices from the S1 vertebra to the dome of the diaphragm. Quantification will be accomplished using a custom MATLAB program that automatically segments visceral and subcutaneous adipose tissue and outputs fat volumes. The program uses morphological and algebraic operators to define a mask of the visceral and subcutaneous cavity. Any voxel consisting of >50% fat will be included in the fat volume. Using this technique, visceral adipose tissue was within 2.4% of the total mean volume for repeat scans in 7 subjects. Hepatic fat will be measured as percent fat content within liver tissue at baseline and wk-6. Six slices will be analyzed, three above and three below the portal vein. To account for variation in lipid content in different regions of the liver, slice by slice analysis will be completed wherein 3 regions of interest (ROI) will be drawn in every lobe of the liver using ImageJ (NIH). Skeletal muscle lipid content will be determined in the quadriceps using the same procedures as described for hepatic fat. Myocardial steatosis and epicardial fat deposition will also be quantified using the same MRI techniques as utilized for abdominal and visceral fat measurements.

3) COGNITIVE AND BEHAVIORAL

a) Cognitive assessment will be done via Automated Neuropsychological Assessment Metrics (ANAM)(Department of Defense, Rockville Pike, MD). This cognitive battery is a library of computer-based tests of domains including attention, concentration, reaction time, memory, processing speed, decision-making, and executive function. The ANAM core testing battery will be used, which assess of the following areas: participant information, sleepiness scale, symptoms checklist, mood scale, simple reaction time, code substitution-learning, procedural reaction time, mathematical processing, matching to sample, code substitution- delayed, simple reaction time (repeated), and go no go.

b) Mood state will be assessed by Profile Of Mood States (POMS) including 37 questions. POMS is a validated measure of psychological distress including a broad range of mental states from anger, confusion, fatigue, to vigor, friendliness.

c) Subjective measures of satiety and hunger will be determined using visual analogue scales. On the same day we test diurnal ketones, we will have

subjects complete these scales to coincide with ketone measures. Additionally, participants will be asked to maintain an event diary to determine diet palatability and any adverse event reporting.

4) **CARDIO-METABOLIC RISK**

A fasting venous blood draw will be collected weekly to analyze serum/plasma:

a) Metabolic panel
b) Lipids (total cholesterol, LDL-C, HDL-C, TG), lipoprotein particle profile
c) Glucose and insulin concentrations to calculate an index of insulin resistance (HOMA-IR). 24-hr urine c-peptide to determine insulin secretion.
d) Inflammatory (CRP, cytokines) and oxidative stress (urine isoprostanes) markers

METHODS

Experimental Approach. This project will be a two-group controlled 6-wk feeding study where all meals are prepared and provided to obese participants (**Fig 1**). All participants will be fed a 25% energy-restricted very low-carbohydrate ketogenic diet designed to induce nutritional ketosis and fat loss. Participants will be randomly assigned to a Ketone Supplement group (*n=12*) who are provided an exogenous pre-formed source of beta-hydroxybutyrate (BOHB) or a Control group (*n=12*) that only receives the standard ketogenic diet. Randomization will be stratified based on insulin resistance, sex, and body composition to ensure balanced group assignment. In addition, we plan to recruit a separate group (n=12) for comparison purposes that will be provided a low-fat diet but otherwise participate in all the same testing as the ketogenic groups. Outcome measurements will be made at various intervals over the 6-wk intervention according to the schedule in **Fig 2**.

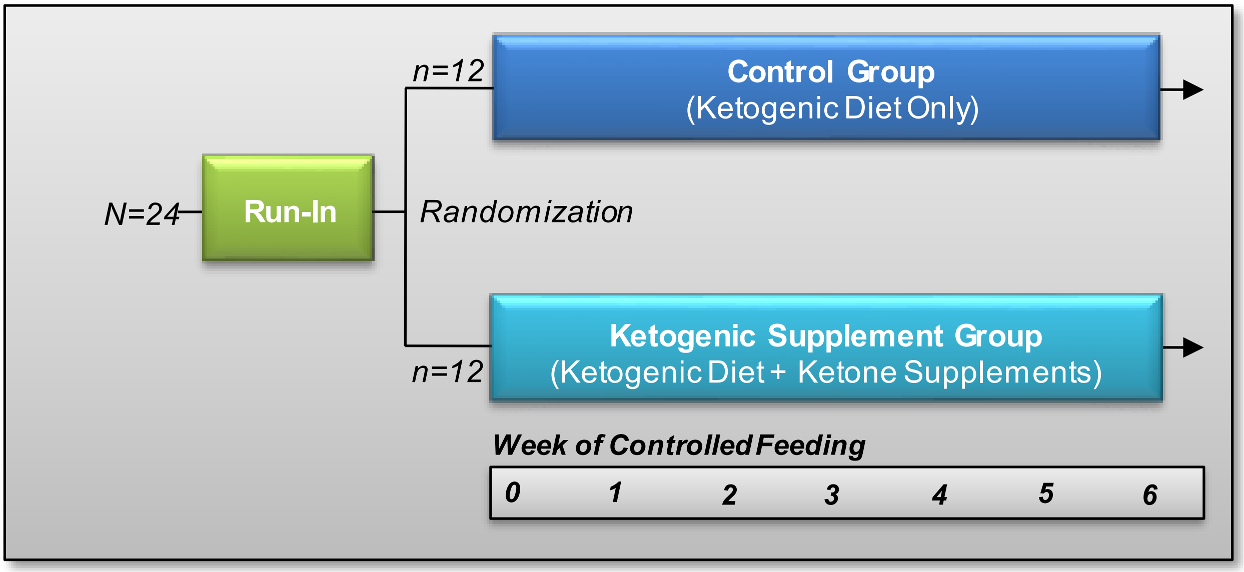

Figure 1. *Experimental approach.*

A table illustrating testing procedures throughout the 6-week intervention is depicted below (**Fig 2**).

Test	Wk 0	Wk 1	Wk 2	Wk 3	Wk 4	Wk 5	Wk 6
Diurnal ketones	X		X		X		X
24-hour urine collection	X		X		X		X
Body Mass	X	X	X	X	X	X	X
Body Composition (DXA)	X		X		X		X
MRI	X						X
Resting Metabolic Rate	X		X		X		X
Fasting Blood Collection	X		X		X		X
Satiety Measures	X	X	X	X	X	X	X
Cognitive Tests	X	X	X		X		X

Figure 2. *Outcome measures*

Rationale for Design. The rationale for the initial 6-wk energy-restricted ketogenic diet is to induce keto-adaptation, the process of switching over to near-exclusive reliance on lipid-based fuels when dietary carbohydrate is restricted. Keto-adaptation is strongly associated with accelerated adipose tissue lipolysis, fatty acid oxidation, and overall fat loss. Thus, the hypocaloric 25% energy-restricted keto-adaptation phase is expected to result in robust improvements in the majority of outcome measures. We expect all participants to show improvements in body composition and decreased ectopic fat depots. Also, the modest energy restriction will induce a steady weight and fat loss, which will increase perceived benefit and motivation of subjects. The addition of exogenous BOHB in the Ketone Supplement group should result in a significant increase in blood ketones. We expect the Control group to reach levels of BOHB between 0.5 - 1.5 mmol/L, on average. Supplementing with exogenous ketones should transiently boost this to the higher end of nutritional ketosis (2.0 to 4.5 mmol/L). A major aim of the project is to determine if the magnitude of change in outcome measures is related to the level of ketosis.

Participants. We will enroll 24 moderately overweight/obese (BMI >27 and ≤35 kg/m^2) men and women aged 21-65 yr. We also plan to enroll an additional 12 subjects to participate in a low-fat comparison group with the same characteristics. All subjects will maintain their same level of activity during the experimental period. Subjects will be informed of the purpose and risks of the investigation prior to signing an informed consent document approved by the IRB. They will also be excluded from participating if they

currently consume a low carbohydrate or ketogenic diet or have experienced >10% weight change in the 6-months preceding their start of the study. Subjects will be excluded if medical screening identifies the participants have diabetes, liver, kidney or other metabolic or endocrine dysfunction, gastrointestinal disorders, are regular smokers, are lactose-intolerant, consume excessive amounts of alcohol (>3 drinks/d or >14 drinks/w). Women who are pregnant or lactating or have initiated or changed their birth control in the past 3 months will also be excluded. Participants taking any probiotics or supplements known to affect serum lipid levels, inflammation, antioxidant status or the gut microbiota will be asked to discontinue use to allow for washout of any metabolic effects. Participants who are already active will be asked to maintain their level of physical activity during the experimental period and sedentary individuals will not be allowed to begin a new exercise program.

This will be the first prospective study to compare responses between a group that receives a ketogenic diet to a group that receives the same ketogenic diet plus ketone supplements. Based on our prior experience with ketogenic diets and acute kinetic studies of ketone supplements, we expect that the dose of ketone supplements used in this project will increase average circulating ketones 50%. We calculate a total sample size of 24 with equal allocation to both groups will be needed to show this magnitude of effect with effect size 1.2, alpha level 0.05 and power 0.8. This sample size will result in varying levels of power for other dependent variables, but as a first exploratory study it will provide important preliminary data on whether certain outcomes related to the keto-adapted phenotype are responsive to ketone supplements.

Participants will be recruited through posted flyers, e-mails, word of mouth, and by using ResearchMatch through the OSU CCTS. Print and email advertisements will instruct interested individuals to call the study center (Volek Lab, Kinesiology Program) for additional information about the study. Participants may either respond to the email address to set up a phone call or may call during the phone-in hours. One of the key personnel involved in the project will describe the study and determine preliminary qualifications by conducting a scripted phone interview. Participant answers to qualifying criteria questions will be recorded to assess whether or not the person calling meets the initial qualifying criteria. If it is determined that the interested participant does meet the initial qualifying criteria, an appointment will be made for the interested participant to visit the study center for a screening meeting. During the screening meeting the study will be described to the interested participant in full detail, an informed consent form will be signed, any questions the interested participant has will be encouraged and responded to and biological data will be collected to determine final eligibility. The data to be collected includes anthropometric measures of height, weight, waist circumference, blood pressure, a pregnancy test for women, a blood draw to assess fasting blood lipids and glucose. A medical history questionnaire, a physical activity questionnaire, a gastrointestinal health screening questionnaire, a dietary questionnaire, a menstrual history questionnaire for women and an MRI readiness questionnaire will all be required to be completed. The MRI readiness questionnaire will determine if the participant can safely have an MRI scan completed. Examples of relevant information include

claustrophobia, previous history of MRI scans and metal implants or devices as outlined by MRIsafety.com.

Recruiting efforts through ResearchMatch will utilize a strategy whereby registered individuals in the ResearchMatch database can be searched for against their non-identifiable volunteer profile in the system. Unidentified individuals meeting search criteria will then be forwarded an electronic recruitment message (see attached *ResearchMatch Recruitment Message*) that identifies them as a potential match for study participation. The secure ResearchMatch clearinghouse will route this standard notification that provides specific study content (i.e. content similar to that of a posted advertisement) to each of these potential ResearchMatch volunteers who will then have the option of replying “yes”, “no”, or not respond through a set of quick links available in this notification. *Note:* This message will not include the study’s direct contact information (e.g. email, phone) as ResearchMatch will measure the response rate through the clearinghouse’s quick links made available in this electronic message. The response rate metrics will be made available to researchers through their ResearchMatch dashboard as well as the Institutional Liaison dashboards. By responding “yes”, the volunteer has authorized ResearchMatch to release their contact information to the researcher responsible for the study. This information will be made available on the researcher’s ResearchMatch study dashboard. The researcher will be responsible for managing this contact information as specified in the IRB-approved study protocol. ResearchMatch will also be collecting aggregate data regarding the status of ResearchMatch volunteers within the study. ResearchMatch volunteers consent to this within the ResearchMatch volunteer agreement. The ResearchMatch enrollment continuum will allow researchers to indicate where the volunteer currently stands within the recruitment process and thus helps researchers monitor the utility and effectiveness of this recruiting tool. Research access to recruit through ResearchMatch will last only as long as the duration of IRB-study approval with the expiration date of ResearchMatch being identical to that of end-date of OSU IRB approval. Researchers will be able to submit current IRB-approval letters for the lifetime of the study and thus provide evidence of successful continuing review applications. If an unintentional lapse in time occurs and the research is not able to submit this continuing review evidence via ResearchMatch, stored ResearchMatch data will not be deleted, but the researcher will not have access to this information until a current IRB-approval letter is uploaded and routed to the Institutional Liaison for review.

Diet Protocol. All food will be purchased, prepared in our research kitchen, and distributed to participants for the 6-wk ketogenic weight loss intervention period. Our research kitchen is ~4,000 sq ft with seven fully functional stations with all the equipment (ovens, work stations, cold storage, utensils, dishwasher/sanitizer systems) and space necessary to receive, store, prepare, and distribute meals. Meals will be packaged and labeled per individual serving sizes and picked up 3-4x/wk. If unable to obtain the food on a particular day, arrangements will be made to ensure the subject has his/her food as planned. No other foods or beverages will be allowed other than very-low-calorie products (e.g., tea, water, diet soda). All food containers will be returned unwashed and uneaten food documented.

We have developed ketogenic meal plans used in prior studies and we are currently conducting 3 studies that use 7-day rotational menus for ketogenic diets. Meals are formulated using typical food items and have been rigorously vetted for palatability and acceptability by research subjects. All participants will consume a hypocaloric ketogenic diet (75% of estimated energy requirements) designed to induced nutritional ketosis. The ketogenic diet will consist of 35 g/day carbohydrate. Normal BOHB levels in a person consuming >100 g/day of carbohydrate are <0.2 mmol/L. The goal of the diet will be to induce a state of nutritional ketosis (BOHB >0.5 mmol/L). The carbohydrate level required to induce nutritional ketosis varies from person to person. Thus, we expect ketones to vary. The diet will be kept constant for all participants over the 6 wk intervention to test how ketones vary among people and between groups. Key aspects of the ketogenic diet are detailed below:

- 25% energy restricted (prior to starting the 6 wk feeding intervention, we will determine daily energy requirements using a combination of information obtained from baseline testing including habitual diet intake, resting metabolic rate, body mass and composition, and formulas (e.g., Harris-Benedict) that consider sex, age, height and activity patterns.
- Carbohydrate: 35 g/day
- Protein: 1.5 g/kg reference weight (determined from midpoint of Metropolitan Height-Weight Tables)
- Fat will comprise the remaining energy intake emphasizing monounsaturated and saturated fat
- 2 servings of medium chain triglyceride (MCT) oil per day (1 serving = 10 g MCT with >90% C8 and C10)

The low-fat comparison group will also be provided all their food at a caloric content the same as the ketogenic groups (i.e., 75% of estimated energy requirements) with the same amount of protein (i.e., 1.5 g/kg reference weight), except the fat and carbohydrate will be different. We will try to mimic the protein sources of the ketogenic menus and adjust the sides to achieve a lower fat and higher carbohydrate. The low-fat diet will consist of ~25% fat, and the remaining calories from carbohydrate (~55% after accounting for protein at ~20%). Added sugars will be kept relatively low (<25 g/day) with a focus on unprocessed carbohydrate-rich foods. Saturated fat will be limited (<10% of total energy) with a focus on unsaturated fat sources. Sodium content will be matched with the ketogenic diet group not receiving the ketone supplement (i.e., ~2300 mg/day).

Supplementation. In addition to the ketogenic diet described above, the Ketone Supplement group will receive 2 servings of exogenous BOHB salts per day. Each serving contains 12 g BOHB and 1874 mg sodium, 570 mg calcium, and 57 mg magnesium. The Control group will receive a placebo with no BOHB or mineral but similar flavoring. Because exogenous BOHB has a caloric value of 4.85 kcal/g, we will decrease the fat content of the Ketone Supplement group by approximately 100 kcal/day to ensure a constant 25% energy restriction for both groups. Supplements will be required to be taken 6 hours apart for each feeding day. The ketone and placebo supplements will be provided by Metagenics, the sponsor of the study. Supplement packets will be provided to subjects

in packets labeled in a manner to maintain the double-blinded nature of the study (e.g., packet A and packet B). We will have the low-fat group consume the Placebo supplement to facilitate comparisons among groups, although investigators will know its contents and thus it will be single-blinded.

Study Procedures

Screening Meeting

Participants that meet the initial qualifying criteria will visit the study center for a screening meeting. The participant and a member of the research team will meet in a private office to discuss the informed consent form. The informed consent form will be provided to the participant for their review, the study will be described in full detail and any questions the interested participant has will be encouraged and responded to. If they choose to participate in the study, they will be asked to sign the consent form providing written consent. The participant will be informed that even though they signed the consent form, their participation in the study is dependent on the results of their blood chemistries, anthropometric measures and questionnaires to determine if they meet the study criteria. If the participant provides consent, they will be provided with a few questionnaires including medical history, physical activity history, a menstrual history for women, a food frequency questionnaire, a gastrointestinal health status questionnaire and an MRI readiness questionnaire. All collected samples will be coded to maintain participant anonymity. We will also measure height, weight and waist circumference. Participants will receive a call from one of the study personnel within one week of the screening visit to inform them if they are eligible or not and will provide the participants with their results from the screening visit. If the participant is eligible for the study and is still interested in participating then he/she will be scheduled to return to the study center for baseline testing.

Baseline Test Session 1. The first test will involve MRI scans to determine visceral fat, liver fat, skeletal muscle fat, myocardial fat, and epicardial fat. The MRI scans will occur at Martha Morehouse Medical Plaza at 2050 Kenny Rd, Columbus, OH 43221 or Ross Heart Hospital at 452 West 10th Ave, Columbus, OH 43210. The total anticipated duration of this testing session is 1 hour.

Assessment of visceral, liver, skeletal muscle, epicardial, and myocardial fat by MRI

Magnetic resonance imaging will be performed using a 3 Tesla MRI system, (MAGNETOM Prisma, Siemens Healthcare, Germany). Subjects will be instructed to sit and then lie down on the MRI table. A chemical shift imaging method will be used that employs multi-echo acquisition to separate fat and water signals. The resulting fat and water images are used to automatically generate a proton density fat fraction map in which the pixel value reflects the percentage of fat within that pixel. From these maps, the fat content of liver tissue, skeletal muscle, and myocardium can be measured. The fat fraction maps are also used to quantify visceral adipose tissue and subcutaneous adipose tissue from abdominal images, and epicardial fat from images of the heart.

For the assessment of liver and abdominal visceral fat the subjects will be asked to hold their breath for the duration of a single scan which lasts about 20s. For the assessment of skeletal muscle fat, the subject will be repositioned in the MRI machine and an additional scan lasting approximately 60 seconds will be run on the thigh muscles. For the assessment of myocardial and epicardial fat, the patient will be repositioned again and a series of 10 to 12 breath-hold scans, each approximately 12 seconds in duration, will be run to cover the heart in the short axis orientation from base to apex. These scans are cardiac-gated and require the application of MRI-safe ECG electrodes to the subject's chest. The ECG signal is used to synchronize the image data acquisition to the beating heart to avoid cardiac motion artifacts.

All of the in vivo image data will be analyzed off-line after the scans are completed. Image analysis will be performed using a combination of software packages. An in-house software application designed to segment and quantify adipose tissue will be used to quantify epicardial fat as well as visceral adipose tissue and abdominal subcutaneous fat. ImageJ software will be used to draw regions of interest and measure fat fraction in the liver, skeletal muscle, and myocardium.

The same MRI scan protocol and data analysis will be repeated at the end of the 6-week feeding period.

Stool Collection. We will collect a stool sample from each subject at baseline and after 6 weeks of intervention to determine differences in microbiota profiles between groups and over time. Each subject will be provided with a sample collection kit comprising sterile collection container, sterile spatula, hygienic tissue, and pair of latex or nitrile gloves. Instructions will be given how to deposit sample into the container. Each sample container will be labeled with numerical ID. After collection the sample container will be returned to the PI laboratory to be frozen and stored. The samples will be homogenized, aliquoted, and DNA preservative will be added to aliquots. About 150 mg of stool will be used for DNA isolation using either QIAamp DNA Stool Mini Kit (Qiagen, Inc) or ZR Stool DNA Isolation Kit (Zymo Research). The kits produce genomic DNA that is free of viable cells. Isolated DNA will be passed through a Zymo-spin IV-HRC filter (Zymo Research) to remove any remaining inhibitors. The isolated genomic DNA will be used for several molecular analyses: 1) microarray analysis with custom microflora array aimed to detect the presence and abundance of 775 different bacterial species in human stools; 2) qPCR tests carried out for the same purpose; 3) molecular analyses of the integrity of isolated genomic DNA using PCR and gel electrophoresis. In these samples, human DNA is considered a contaminant, is often degraded during subsequent procedures, and will never be used for any gene/genome analysis. In a separate procedure, an aliquot of fecal contents (~200mg) will be added to sterile PBS. The mixture will be vortexed to homogeneity. The resulting solution will be centrifuged at 13,000 rpm in the refrigerated microcentrifuge to pellet down any remaining particles, and the supernatant will be

retained. This “fecal water” will be used later in NMR experiments to profile short chain fatty acid composition of stool samples.

Baseline Test Session 2. The first part of this testing session will occur in the Physical Activity and Educational Services (PAES) building on The Ohio State University campus. Subjects will be asked to continue performing finger sticks and collecting urine once they leave the laboratory (explained later). The address for the PAES building is 305 West 17th Ave, Columbus, OH 43210.

This testing session will represent the first day of the ketogenic diet (or low-fat diet if in the Low-Fat group). Prior to this test session the participants need to be fasted overnight, which means the participant cannot consume any food or drink other than water for 10-12 hours prior to the session. To ensure proper hydration, participants will be asked to consume 2 cups of water the night before the session and another 2 cups the morning of the session. The following in-lab tests will occur as outlined in **Fig 3**.

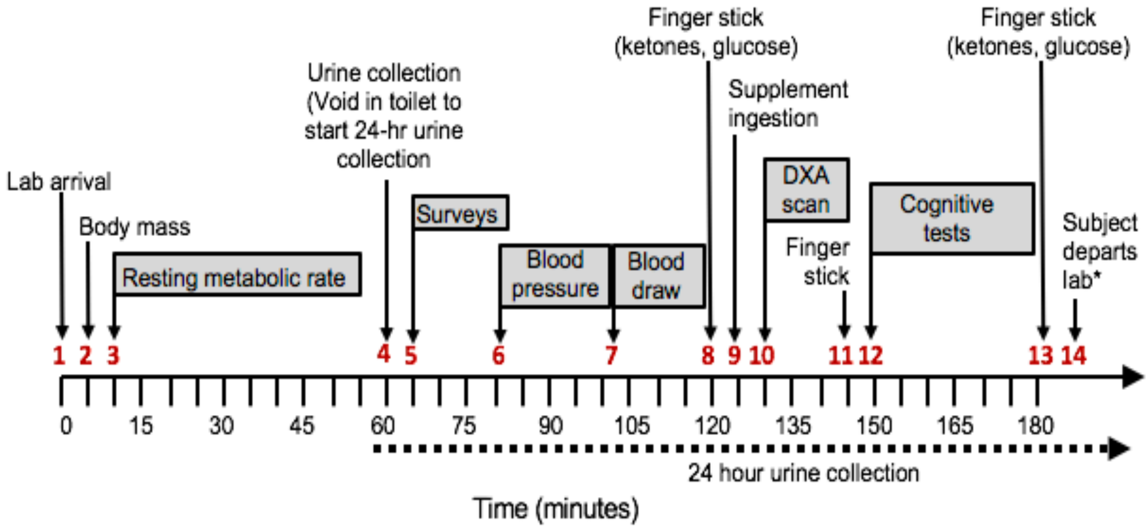

Figure 3. *Test session details.* *Subject departs lab with: 1) meter/strips to perform hourly finger sticks for next 10-hr (12-hr total), 2) container to collect urine for 24-hr, 3) standardized food to be consumed at specific times, 4) supplements to be consumed at specific times, 5) surveys to document hunger/satiety.

1. **Lab arrival**: Subjects arrived fasted and hydrated. Diet and exercise will be controlled the day before.
2. **Body mass:** Height and body mass will be measured.
3. **Resting metabolic rate:** Resting energy metabolism will be measured to determine how many calories are expended at rest. This test requires that participants lie still and relax for 30 min. The room will be dark, quiet and at a comfortable temperature.

After a 30 min rest period the participant will remain awake but still. A hood will be placed over their head so they can breathe normally while we collect samples of the air being inhaled and exhaled. A metabolic cart attached to the hood via a plastic hose will measure expired gases to determine energy expenditure and substrate use.

4. **Urine collection:** We will ask the participant to void into the toilet as much urine as possible and mark the time. For the following 24-hr period participants will urinate into containers that will be provided. When the participant wakes up the next morning exactly 24-hr later they will be instructed to force as much urine out as possible into the container.

5. **Surveys:** Subjects will complete a survey to evaluate psychological components of mood, resiliency, and stress. The Profile Of Mood States (POMS) survey has 37 questions and is validated as a measure of psychological distress including a broad range of mental states from anger, confusion, fatigue, to vigor, friendliness. Subjects will also complete a visual analogue mood scale (VAMS) to determine eight specific mood states (e.g., afraid, confused, sad, angry, energetic, tired, happy, and tense). Subjects will also complete questionnaires asking about digestive health, physical activity and recent lifestyle changes.

6. **Blood pressure.** Resting blood pressure will be measured 3 times, 5 min apart in each arm by auscultation and averaged.

7. **Blood draw:** Blood will be drawn from an arm vein using a small needle. A total of 47 mL (~3 T) will be taken.

8. **Finger stick:** Using a lancet device a small amount of capillary blood will be obtained from a fingertip for immediate determination of beta-hydroxybutyrate and glucose concentration. From this point on, finger sticks will be made every hour for the next 12-hours.

   In addition to measuring ketones in your blood from the finger stick, we will measure another type of ketone (acetone) in your breath. This test will be done while you are in the lab performing other tests as described in the figure on the next page. All that is required during this test is to take a breath and then 'blow' firmly into a breathing tube that will be connected to a device that measures ketones. We will have you blow at least twice into the tube on three separate occasions during the time you will be in the lab.

9. **Supplement ingestion:** Participants will ingest a supplement corresponding to their group assignment.

10. **DXA scan:** Body composition and bone density will be determined using dual-energy X-ray absorptiometry (DXA). Subjects will lie quietly on the DXA bed while a scanning arm passes over their body from head to toe. The scan takes about 7 min. A certified technician will perform the scan. In addition to the DXA scan for body composition, the circumference of the participant's waist will be measured using a standard tape measurer.

11. **Finger stick:** In addition to the hourly finger sticks, we will measure beta-hydroxybutyrate and glucose 20 min after supplement ingestion to determine acute postprandial effects on ketosis and glycemia.

12. **Cognitive Tests.** Participants will perform the Automated Neuropsychological Assessment Metrics (ANAM) mental test battery to determine changes in cognitive function. For the ANAM battery, participants will sit in a chair and be given a touch screen computer/tablet to perform a series of tests as instructed. These tests are designed to tests attention, concentration, mental reaction time, memory, mental processing speed, and decision-making.

13. **Finger stick:** Another 1-hr finger stick will be performed to determine beta-hydroxybutyrate and glucose.

14. **Subject departs lab:** Participants will be provided detailed instructions and supplies to continue hourly measurements of beta-hydroxybutyrate and glucose over the next 12-hr and urine collection over 24-hr. Food and supplements will be provided with instructions to come at specific times. Satiety surveys will be completed six times during the course of the day.

The entire series of lab tests on this day will take approximately 3 to 3.5-hr. This test session will be repeated at 2, 4 and 6 weeks of the study. The same diet will be consumed the day before and the day of this test session.

After the subjects depart the lab, they will continue the hourly finger sticks for a total of 12 hours. Supplements will be required to be taken 6 hours apart, and meals will be eaten every 4 hours. Participants will be asked to record all non-caloric beverages in order that they may be standardized on subsequent testing days. Urine collection will continue throughout the day. The specific details for the out-of-lab testing portion are depicted below in **Fig 4**.

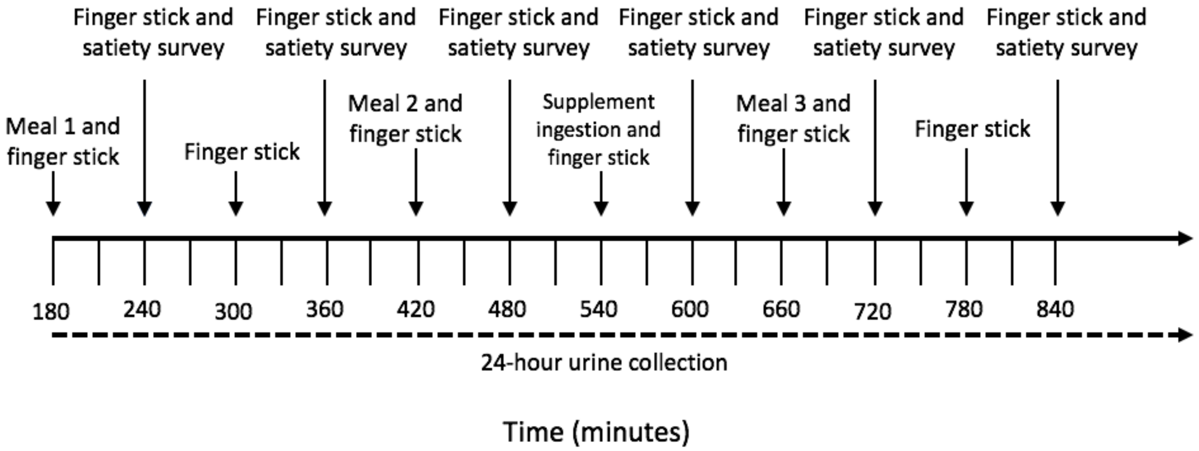

Figure 4. *Out of lab test day details.*

Privacy/Confidentiality

For all the data collected over the course of the study for each participant (i.e. records, biological samples and questionnaires) a unique subject identifier (i.e. a code) will be assigned and used instead of the subject's name. The code for each participant which links the subject name with their identifier will only be available to research personnel. Any records that contain the subject's name and identifier will either be stored in the Kinesiology file storage room in a file cabinet (locked) or protected on a computer via password protection on the individual digital file and password protection on the computer the file(s) are stored on. All other records that contain the subject identifier only will also be kept in either a file cabinet in our locked file storage room or on a password protected computer. Subject names will never be used in any presentation or publication resulting from this study. The records will be maintained until the data are published and up to a maximum of ten years after the completion of the study.

Prior to signing the informed consent form all participants will be informed that their participation in the study is voluntary and that they may withdraw at any time. Participants who provide written consent are considered to be enrolled in the study. All records or biological data obtained after signing of the informed consent (including the screening visit, even for subjects that are not eligible for participation in the study) are treated with the same confidentiality safety measures as those subjects who qualify. Any information obtained during the prescreening for participants that were not eligible will be deleted.

References

1. Owen OE, et al. Brain Metabolism during Fasting. *J Clin Invest*. 1967;46(10):1589-1595.
2. Newman JC, Verdin E. β-hydroxybutyrate: Much more than a metabolite. *Diabetes Res Clin Pract*. 2014;106(2):173-181.
3. Volek JS, et al. Rethinking fat as a fuel for endurance exercise. Eur J Sport Sci. 2015;15(1):13-20.
4. Volek JS, et al. Dietary carbohydrate restriction induces a unique metabolic state positively affecting atherogenic dyslipidemia, fatty acid partitioning, and metabolic syndrome. Prog Lipid Res. 2008 Sep;47(5):307-18.
5. Feinman RD, et al. Dietary carbohydrate restriction as the first approach in diabetes management: critical review and evidence base. Nutrition. 2015 Jan;31(1):1-13.
6. Branco AF, et al. Ketogenic diets: from cancer to mitochondrial diseases and beyond. Eur J Clin Invest. 2016 Mar;46(3):285-98.
7. Shimazu T, et al. Suppression of oxidative stress by β-hydroxybutyrate, an endogenous histone deacetylase inhibitor. *Science*. 2013;339(6116):211-214.
8. Volek JS, et al. Carbohydrate restriction has a more favorable impact on the metabolic syndrome than a low fat diet. Lipids. 2009 Apr;44(4):297-309.
9. Volek JS, et al. Metabolic characteristics of keto-adapted ultra-endurance runners. Metabolism. 2016 Mar;65(3):100-10.
10. McKenzie AL, et al. Novel Intervention including Individualized Nutritional Recommendations Reduces HbA1c, Medication Use, and Weight in Type-2 Diabetes.

JMIR Diabetes. 2017; 2(1):e5.
