# Supplementary material for: Self-reported menses physiology is positively modulated by a well-formulated, energy-controlled ketogenic diet vs. low fat diet in women of reproductive age with overweight/obesity
Source: PLoS One. 2024 Aug 16;19(8):e0293670. doi: 10.1371/journal.pone.0293670 (PMC11329152; doi:10.1371/journal.pone.0293670)
Supplement: S3 File — (DOCX) [file pone.0293670.s003.docx]

| **ST 1.** Nutrient Composition of Experimental Diets | | | |
| --- | --- | --- | --- |
| **Variable** | **KD+KS** | **KD+PL** | **LFD** |
| Energy (kcal/day) | 1845 (408) | 1752 (350) | 1900 (296) |
| Protein (g) | 99 (12) | 100 (10) | 100 (8) |
| Carbohydrate (g) | 40 (4) | 38 (3) | 259 (48) |
| Sugar (g) | 17 (2) | 17 (2) | 101 (20) |
| Fiber (g) | 10 (1) | 10 (1) | 34 (5) |
| Added Sugars (g) | n/a | n/a | <25g/day |
| Fat (g) | 143 (39) | 131 (4) | 51 (9) |
| SFA (g) | 63 (16) | 63 (15) | 17 (10) |
| MUFA (g) | 38 (12) | 38 (11) | 10 (2) |
| PUFA (g) | 8 (3) | 8 (3) | 7 (3) |
| Cholesterol (g) | 414 (119) | 402 (106) | 154 (10) |
| Sodium (mg) | 6100 (56) | 2351 (82) | 1974 (163) |
| Potassium (mg) | 2211 (208) | 2243 (175) | 2758 (350) |
| Calcium (mg) | 2001 (156) | 880 (126) | 1008 (71) |
| *Values reported as mean (SD)* | | | |
| *SFA= saturated fatty acids, MUFA = monounsaturated fatty acids; PUFA= polyunsaturated fatty acids* | | | |

| **ST 2. P**redictive baseline characteristics between reported change in menses vs. no change in menses. | | | | | | |
| --- | --- | --- | --- | --- | --- | --- |
| **Baseline Variables** | **Change in Menses (*n* = 8)** | | **No Change in Menses (*n* = 11)** | | **Independent**  **t-test** | **Effect**  **Size** |
|  | Mean | SD | Mean | SD | *p-*value | Cohen's *d* |
| Body Weight (kg) | 87.18 | (8.44) | 84.76 | (10.15) | 0.578 | 0.26 |
| BMI (kg/m2) | 32.71 | (2.78) | 30.20 | (1.84) | **0.040** | 1.03 |
| DXA Fat Mass (kg) | 36.53 | (6.92) | 33.45 | (5.69) | 0.317 | 0.48 |
| DXA Fat-Free Mass (kg) | 46.86 | (2.87) | 48.48 | (6.40) | 0.465 | 0.35 |
| DXA Body Fat Percentage (%) | 42% | 5% | 39% | 4% | 0.194 | 0.63 |
| Fasting Glucose (mg/dL) | 85.9 | (15.5) | 78.1 | (13.9) | 0.276 | 0.52 |
| Fasting Insulin (μU/mL) | 11.04 | (4.07) | 14.76 | (7.86) | 0.195 | 0.63 |
| HOMA-IR | 2.36 | (1.01) | 2.91 | (1.80) | 0.407 | 0.40 |
| Total Cholesterol (mg/dL) | 189.18 | (33.60) | 181.13 | (30.94) | 0.601 | 0.25 |
| HDL-C (mg/dL) | 58.82 | (21.04) | 56.25 | (19.10) | 0.788 | 0.13 |
| Triglycerides (mg/dL) | 118.64 | (66.73) | 119.00 | (86.79) | 0.992 | 0.00 |
| LDL-C (mg/dL) | 99.73 | (17.29) | 103.25 | (22.48) | 0.704 | 0.18 |
| Total Cholesterol: HDL-C Ratio | 3.41 | (1.19) | 3.56 | (1.47) | 0.804 | 0.12 |
| non-HDL-C (mg/dL) | 121.3 | (20.13) | 124.9 | (30.79) | 0.761 | 0.14 |
| IL-1β (pg/mL) | 0.27 | (0.17) | 0.19 | (0.08) | 0.214 | 0.60 |
| IL-6 (pg/mL) | 2.32 | (2.51) | 0.99 | (0.40) | 0.158 | 0.69 |
| IL-8 (pg/mL) | 7.34 | (2.25) | 7.73 | (3.02) | 0.751 | 0.15 |
| IL-10 (pg/mL) | 0.44 | (0.24) | 0.53 | (0.32) | 0.483 | 0.33 |
| MCP-1 (pg/mL) | 96.62 | (23.83) | 78.06 | (13.14) | 0.064 | 0.92 |
| TNF-α (pg/mL) | 2.78 | (1.20) | 2.98 | (1.07) | 0.714 | 0.17 |

| **ST 3.** Baseline Comparison Between Regain of Menses Within-KD and Compared to the LFD. | | | | | | | |
| --- | --- | --- | --- | --- | --- | --- | --- |
| **Baseline Variables** | **Regain KD (*n* = 6)** | | **No Regain KD (*n* = 7)** | | **LFD (*n* = 6)** | | **One-Way ANOVA** |
|  | Mean | SD | Mean | SD | Mean | SD | *p-*value |
| Body Weight (kg) | 86.53 | (8.63) | 84.97 | (9.26) | 87.18 | (10.55) | 0.911 |
| BMI (kg/m^2^) | 32.63 | (2.90) | 32.09 | (2.71) | 30.16 | (2.17) | 0.255 |
| DXA Fat Mass (kg) | 37.21 | (7.67) | 34.98 | (5.90) | 33.56 | (6.41) | 0.639 |
| DXA Fat-Free Mass (kg) | 45.64 | (2.98) | 46.46 | (3.64) | 50.71 | (5.78) | 0.116 |
| DXA Body Fat Percentage (%) | 43% | (6%) | 41% | (3%) | 38% | (4%) | 0.145 |
| Fasting Glucose (mg/dL) | 86.3 | (21.8) | 84.4 | (4.1) | 76.8 | (15.9) | 0.532 |
| Fasting Insulin (μU/mL) | 11.11 | (2.99) | 9.63 | (5.03) | 17.58 | (6.95) | **0.037** |
| HOMA-IR | 2.42 | (1.06) | 2.01 | (0.99) | 3.45 | (1.77) | 0.163 |
| Total Cholesterol (mg/dL) | 199.83 | (34.45) | 171.86 | (31.52) | 188.00 | (27.66) | 0.298 |
| HDL-C (mg/dL) | 50.83 | (14.36) | 67.57 | (22.53) | 53.17 | (19.21) | 0.259 |
| Triglycerides (mg/dL) | 104.33 | (28.55) | 119.14 | (84.30) | 132.83 | (98.07) | 0.816 |
| LDL-C (mg/dL) | 111.67 | (9.00) | 83.57 | (13.53) | 111.33 | (18.06) | **0.003** |
| Total Cholesterol: HDL-C Ratio | 3.88 | (1.07) | 2.73 | (0.98) | 3.93 | (1.53) | 0.151 |
| non-HDL-C (mg/dL) | 132.3 | (10.50) | 104.3 | (20.49) | 134.8 | (27.80) | **0.030** |
| IL-1β (pg/dL) | 0.23 | (0.06) | 0.28 | (0.21) | 0.18 | (0.09) | 0.439 |
| IL-6 (pg/dL) | 1.39 | (1.09) | 2.85 | (2.94) | 0.86 | (0.36) | 0.177 |
| IL-8 (pg/dL) | 7.65 | (1.47) | 7.37 | (2.77) | 7.51 | (3.40) | 0.983 |
| IL-10 (pg/dL) | 0.40 | (0.18) | 0.46 | (0.26) | 0.58 | (0.37) | 0.538 |
| MCP-1 (pg/dL) | 98.22 | (17.67) | 91.32 | (27.37) | 76.46 | (13.69) | 0.213 |
| TNF-α (pg/dL) | 2.69 | (1.14) | 2.92 | (1.20) | 2.97 | (1.19) | 0.911 |

| **ST 4 \| Six-Week Results (*n* = 19)** | | | | | | | | | | |
| --- | --- | --- | --- | --- | --- | --- | --- | --- | --- | --- |
|  | **Timepoint** | **KD+KS** | | **KD+PL** | | **LFD** | | **3 x 4 RM ANOVA (*p*-values)** | | |
|  |  | Mean | SD | Mean | SD | Mean | SD | Condition | Time | Interaction |
| Bodyweight  (kg) | Day 1 | 80.4 | (2.0) | 90.2 | (9.7) | 87.2 | (10.5) | 0.171 | **<0.001** | 0.16 |
|  | Day 14 | 77.0 | (2.2) | 85.7 | (9.5) | 84.3 | (10.1) |  |  |  |
|  | Day 28 | 75.6 | (2.6) | 84.1 | (9.6) | 81.8 | (9.2) |  |  |  |
|  | Day 42 | 74.4 | (2.6) | 82 | (9.3) | 80.4 | (9.1) |  |  |  |
| BMI  (kg/m^2^) | Day 1 | 31.3 | (1.2) | 33.2 | (3.4) | 30.2 | (2.2) | 0.182 | **<0.001** | 0.164 |
|  | Day 14 | 30.0 | (1.4) | 31.6 | (3.4) | 29.1 | (2.0) |  |  |  |
|  | Day 28 | 29.5 | (1.7) | 31 | (3.4) | 28.3 | (1.7) |  |  |  |
|  | Day 42 | 29.0 | (1.6) | 30.2 | (3.5) | 27.8 | (1.7) |  |  |  |
| DXA Fat Mass  (kg) | Day 1 | 32.7 | (3.9) | 38.9 | (7.3) | 33.6 | (6.4) | 0.149 | **<0.001** | 0.777 |
|  | Day 14 | 31.1 | (4.0) | 37.3 | (7.2) | 31.8 | (6.1) |  |  |  |
|  | Day 28 | 29.6 | (3.9) | 35.8 | (7.4) | 29.8 | (6.3) |  |  |  |
|  | Day 42 | 28.0 | (4.4) | 34.5 | (7.4) | 28.8 | (5.7) |  |  |  |
| DXA Fat-Free Mass (kg) | Day 1 | 44.8 | (3.3) | 47.2 | (2.9) | 50.7 | (5.8) | 0.101 | **<0.001** | 0.715 |
|  | Day 14 | 43.6 | (2.9) | 45.9 | (3.5) | 49.4 | (5.8) |  |  |  |
|  | Day 28 | 43.8 | (3.6) | 45.9 | (3.8) | 49.2 | (5.3) |  |  |  |
|  | Day 42 | 43.7 | (4.2) | 45.2 | (4.0) | 48.7 | (5.1) |  |  |  |
| DXA Body Fat Percentage (%) | Day 1 | 40.3% | 4.6% | 43.4% | 4.8% | 43.4% | 4.8% | 0.097 | **0.016** | 0.609 |
|  | Day 14 | 38.9% | 4.8% | 42.2% | 4.9% | 42.2% | 4.9% |  |  |  |
|  | Day 28 | 37.7% | 5.6% | 41.5% | 5.0% | 41.5% | 5.0% |  |  |  |
|  | Day 42 | 35.9% | 4.2% | 38.8% | 4.6% | 38.8% | 4.6% |  |  |  |
| Ketones  (*R*-βHB) | Day 1 | 0.3 | (0.1) | 0.2 | (0.1) | 0.1 | (0.1) | **0.014** | **<0.001** | **0.032** |
|  | Day 14 | 1.7^#^ | (1.2) | 1.7^#^ | (1.0) | 0.3 | (0.1) |  |  |  |
|  | Day 28 | 1.5^#^ | (1.2) | 1.1^#^ | (0.7) | 0.3 | (0.1) |  |  |  |
|  | Day 42 | 1.3^#^ | (1.2) | 1.2^#^ | (0.4) | 0.2 | (0.1) |  |  |  |
| Fasting Glucose (mg/dL) | Day 1 | 87.7 | (9.0) | 83.3 | (18.4) | 76.8 | (15.9) | 0.654 | 0.587 | 0.476 |
|  | Day 14 | 79.3 | (12.4) | 74.1 | (11.4) | 82.7 | (5.5) |  |  |  |
|  | Day 28 | 82.7 | (9.4) | 82.7 | (8.8) | 79.8 | (4.0) |  |  |  |
|  | Day 42 | 83.0 | (10.0) | 77.6 | (12.4) | 79.0 | (11.2) |  |  |  |
| Fasting Insulin  (μU/mL) ^‡^ | Day 1 | 8.4 | (1.9) | 12 | (4.9) | 17.6^†^ | (6.9) | 0.216 | **<0.001** | **0.003** |
|  | Day 14 | 7.1 | (3.9) | 5.7 | (4.3) | 7.9 | (2.3) |  |  |  |
|  | Day 28 | 5.9 | (1.3) | 7.3 | (5.6) | 8.2 | (3.8) |  |  |  |
|  | Day 42 | 8.4 | (4.4) | 6.6 | (4.5) | 10.4 | (4.4) |  |  |  |
| HOMA-IR | Day 1 | 1.8 | (0.5) | 2.5 | (1.3) | 3.5 | (1.8) | 0.423 | **<0.001** | 0.062 |
|  | Day 14 | 1.4 | (1.0) | 1.1 | (0.9) | 1.6 | (0.4) |  |  |  |
|  | Day 28 | 1.2 | (0.4) | 1.5 | (1.2) | 1.6 | (0.7) |  |  |  |
|  | Day 42 | 1.8 | (1.1) | 1.4 | (1.1) | 2.0 | (0.8) |  |  |  |
| Total Cholesterol (mg/dL) | Day 1 | 179.3 | (24.3) | 189.4 | (43.1) | 188 | (27.7) | 0.309 | **<0.001** | 0.089 |
|  | Day 14 | 173.6 | (27.9) | 160.3 | (27.8) | 139.8 | (36.9) |  |  |  |
|  | Day 28 | 170.7 | (28.5) | 158.3 | (27.6) | 135.8 | (32.5) |  |  |  |
|  | Day 42 | 166.2 | (37.2) | 170.3 | (24.0) | 135.3 | (35.1) |  |  |  |
| HDL-C  (mg/dL) | Day 1 | 60.7 | (19.6) | 59.1 | (22.6) | 53.2 | (19.2) | 0.327 | **0.002** | 0.495 |
|  | Day 14 | 57.5 | (21.4) | 52.7 | (17.7) | 41.7 | (11.9) |  |  |  |
|  | Day 28 | 53.5 | (19.3) | 49.6 | (14.5) | 38.5 | (8.9) |  |  |  |
|  | Day 42 | 50.7 | (16.9) | 54.1 | (18.7) | 37 | (8.9) |  |  |  |
| Triglycerides  (mg/dL) | Day 1 | 120.8 | (70.3) | 105 | (60.4) | 132.8 | (98.1) | 0.832 | **0.028** | 0.406 |
|  | Day 14 | 106.4 | (44.4) | 91.4 | (37.5) | 64.0 | (19.2) |  |  |  |
|  | Day 28 | 89.8 | (26.5) | 86.0 | (39.8) | 77.7 | (38.7) |  |  |  |
|  | Day 42 | 91.7 | (16.5) | 83.7 | (29.6) | 84.2 | (36.3) |  |  |  |
| LDL-C  (mg/dL) | Day 1 | 97.3 | (22.8) | 95.9 | (15.5) | 111.3 | (18.1) | 0.786 | **0.015** | **0.013** |
|  | Day 14 | 96.7 | (22.1) | 89.2 | (22.3) | 83.0 | (32.2) |  |  |  |
|  | Day 28 | 98.8 | (19.1) | 90.9 | (22.0) | 80.8 | (27.3) |  |  |  |
|  | Day 42 | 97.0 | (31.1) | 98.4 | (22.7) | 80.7 | (24.8) |  |  |  |
| Total Cholesterol: HDL-C Ratio | Day 1 | 3.2 | (1.2) | 3.3 | (1.2) | 3.9 | (1.5) | 0.806 | 0.691 | 0.607 |
|  | Day 14 | 3.4 | (1.1) | 3.3 | (0.8) | 3.6 | (1.5) |  |  |  |
|  | Day 28 | 3.5 | (1.2) | 3.4 | (0.9) | 3.7 | (1.3) |  |  |  |
|  | Day 42 | 3.6 | (1.2) | 3.4 | (1.1) | 3.7 | (1.0) |  |  |  |
| non-HDL-C  (mg/dL) | Day 1 | 118.7 | (23.3) | 116 | (21.9) | 134.8 | (27.8) | 0.791 | **0.003** | **0.011** |
|  | Day 14 | 116.1 | (20.6) | 107.5 | (24.7) | 98.2 | (34.2) |  |  |  |
|  | Day 28 | 117.2 | (20.5) | 108.7 | (26.4) | 97.3 | (30.1) |  |  |  |
|  | Day 42 | 115.5 | (31.9) | 116.1 | (26.6) | 98.3 | (30.2) |  |  |  |
| IL-1β  (pg/mL) | Day 1 | 0.31 | (0.19) | 0.21 | (0.10) | 0.18 | (0.09) | **0.003** | 0.536 | 0.844 |
|  | Day 14 | 0.29 | (0.12) | 0.14 | (0.04) | 0.14 | (0.10) |  |  |  |
|  | Day 28 | 0.21 | (0.06) | 0.16 | (0.08) | 0.18 | (0.12) |  |  |  |
|  | Day 42 | 0.28 | (0.19) | 0.17 | (0.07) | 0.14 | (0.04) |  |  |  |
| IL-6  (pg/mL) | Day 1 | 1.25 | (1.11) | 2.97 | (2.86) | 0.86 | (0.36) | 0.075 | 0.210 | 0.232 |
|  | Day 14 | 1.23 | (0.60) | 1.14 | (0.64) | 0.70 | (0.23) |  |  |  |
|  | Day 28 | 1.25 | (1.02) | 1.17 | (0.61) | 0.89 | (0.49) |  |  |  |
|  | Day 42 | 1.18 | (0.59) | 1.22 | (0.67) | 0.86 | (0.35) |  |  |  |
| IL-8  (pg/mL) | Day 1 | 6.81 | (1.08) | 8.09 | (2.77) | 7.51 | (3.40) | 0.896 | 0.447 | 0.161 |
|  | Day 14 | 9.13 | (3.92) | 7.72 | (2.62) | 7.33 | (2.10) |  |  |  |
|  | Day 28 | 7.19 | (1.98) | 7.41 | (2.43) | 7.27 | (1.35) |  |  |  |
|  | Day 42 | 6.06 | (2.46) | 8.09 | (2.69) | 7.73 | (2.30) |  |  |  |
| IL-10  (pg/mL) | Day 1 | 0.28 | (0.13) | 0.56 | (0.19) | 0.58 | (0.37) | 0.091 | 0.158 | 0.093 |
|  | Day 14 | 0.29 | (0.10) | 0.53 | (0.29) | 0.48 | (0.33) |  |  |  |
|  | Day 28 | 0.28 | (0.10) | 0.43 | (0.13) | 0.58 | (0.28) |  |  |  |
|  | Day 42 | 0.31 | (0.12) | 0.51 | (0.17) | 0.68 | (0.42) |  |  |  |
| MCP-1  (pg/mL) | Day 1 | 98.4 | (18.9) | 91.2 | (26.6) | 76.5 | (13.7) | 0.073 | 0.825 | 0.257 |
|  | Day 14 | 111.6 | (10.7) | 93.5 | (31.4) | 66.8 | (11.9) |  |  |  |
|  | Day 28 | 103.2 | (30.5) | 89.3 | (24.5) | 74.1 | (15.3) |  |  |  |
|  | Day 42 | 100.7 | (26.4) | 90.4 | (26.3) | 81.7 | (18.2) |  |  |  |
| TNF-α  (pg/mL) | Day 1 | 2.16 | (0.82) | 3.38 | (1.09) | 2.97 | (1.19) | 0.133 | 0.176 | **0.001** |
|  | Day 14 | 2.38 | (0.69) | 2.88 | (0.56) | 2.84 | (1.17) |  |  |  |
|  | Day 28 | 2.19 | (0.47) | 2.95 | (0.84) | 3.28 | (1.04) |  |  |  |
|  | Day 42 | 2.17 | (0.47) | 2.97 | (0.77) | 3.73 | (1.32) |  |  |  |
| ^#^ = *p* < 0.05 compared to the LFD | | | | | | | | | | |
| ^†^ = *p* < 0.05 greater than the KD+KS and KD+PL at Day 1. | | | | | | | | | | |
| ^‡^ = An ANCOVA model was used to analyze these results. | | | | | | | | | | |
| KD, ketogenic diet; KS, ketone salts; PL, placebo; LFD, low-fat diet; BMI, body mass index; DXA, dual x-ray absorptiometry; FM = fat mass; FFM, fat-free mass; BF, body fat percentage; HOMA-IR, homeostatic model of insulin resistance; TC, total cholesterol; TG, triglycerides; HDL-/LDL-C, calculated high/low-density lipoprotein; IL, interleukin; MCP, monocyte chemoattractant protein; TNF, tumor necrosis factor. | | | | | | | | | | |
| HOMA-IR = [glucose (mg/dL)] * [insulin(µU/L)] / 405. | | | | | | | | | | |

**SF 1. Weight-loss and Body Composition Changes.**

Data presented as mean ± SD.

Main effect: **, *** = *p* < 0.01, 0.001 from Day 1

**SF 2. Blood Panel.**

Data presented as mean ± SD.

Main effect: *** = *p* < 0.001 from Day 1**.**
