# Supplementary material for: Self-reported menses physiology is positively modulated by a well-formulated, energy-controlled ketogenic diet vs. low fat diet in women of reproductive age with overweight/obesity
Source: PLoS One. 2024 Aug 16;19(8):e0293670. doi: 10.1371/journal.pone.0293670 (PMC11329152; doi:10.1371/journal.pone.0293670)
Supplement: Attachment — Submitted filename: rev. one plose.docx [file pone.0293670.s005.docx]

Dear authors

It is a very good and valuable work, but if the following points are included in, it will be a very good paper.

-The title doesn't match with the text. Because in your title, there is no mention of ketone supplementation and compared with a low fat diet (LFD).

- The necessity and objectives of the research are not well stated at the end of the introduction, so there should be more discussion about supplementation and why it is used during menstruation.

- In the methods section, the method of prescribing and controlling diets must be described in more detail. How is ketosis status controlled?

-The composition of macronutrients and total energy in each diet plan better to be mentioned and compared in a table.

-Which reference was used for the Menses Survey? Is it valid? Please give a reference.

-It is better to be mentioned the Menses stages by the participants at the time of evaluation
