# Supplementary material for: Self-reported menses physiology is positively modulated by a well-formulated, energy-controlled ketogenic diet vs. low fat diet in women of reproductive age with overweight/obesity
Source: PLoS One. 2024 Aug 16;19(8):e0293670. doi: 10.1371/journal.pone.0293670 (PMC11329152; doi:10.1371/journal.pone.0293670)
Supplement: Attachment — Submitted filename: Response to Reveiwers.docx [file pone.0293670.s006.docx]

Dear Editor and Reviewers,

Thank you for your expertise and critique of our manuscript. We found your suggestions and requests extremely helpful. We have provided our answers and changes to each comment below in red.

Reviewer Comments:

1. Please ensure that your manuscript meets PLOS ONE's style requirements, including those for file naming. The PLOS ONE style templates can be found at

https://journals.plos.org/plosone/s/file?id=wjVg/PLOSOne_formatting_sample_main_body.pdf and <https://journals.plos.org/plosone/s/file?id=ba62/PLOSOne_formatting_sample_title_authors_affiliations.pdf>

Thank you. We have edited our manuscript using the provided formatting template.

2. We note that the grant information you provided in the ‘Funding Information’ and ‘Financial Disclosure’ sections do not match.

When you resubmit, please ensure that you provide the correct grant numbers for the awards you received for your study in the ‘Funding Information’ section.

We have included the grant award details.

3. Thank you for stating the following in the Competing Interests section:

"JV receives royalties for low-carbohydrate nutrition books; is founder, consultant, and stockholder of Virta Health, Inc. and is a member of the advisory boards for Simply Good Foods. The remaining authors declare that the research was conducted in the absence of any commercial or financial relationships that could be construed as a potential conflict of interest."

Please confirm that this does not alter your adherence to all PLOS ONE policies on sharing data and materials, by including the following statement: "This does not alter our adherence to PLOS ONE policies on sharing data and materials.” (as detailed online in our guide for authors http://journals.plos.org/plosone/s/competing-interests). If there are restrictions on sharing of data and/or materials, please state these. Please note that we cannot proceed with consideration of your article until this information has been declared.

We confirm our adherence to all PLOS ONE policies.

Please include your updated Competing Interests statement in your cover letter; we will change the online submission form on your behalf.

No changes to this section.

4. Please include captions for your Supporting Information files at the end of your manuscript, and update any in-text citations to match accordingly. Please see our Supporting Information guidelines for more information: http://journals.plos.org/plosone/s/supporting-information.

Thank you, for bringing this to light. This has been added.

5. We note you have included a table to which you do not refer in the text of your manuscript. Please ensure that you refer to Table 2 in your text; if accepted, production will need this reference to link the reader to the Table.

Thank you for bringing this to our attention. Table 2 has been referenced.

6. Please ensure that you refer to Figure 3 in your text as, if accepted, production will need this reference to link the reader to the figure

Figure 3 has been referenced.

7. Please review your reference list to ensure that it is complete and correct. If you have cited papers that have been retracted, please include the rationale for doing so in the manuscript text, or remove these references and replace them with relevant current references. Any changes to the reference list should be mentioned in the rebuttal letter that accompanies your revised manuscript. If you need to cite a retracted article, indicate the article’s retracted status in the References list and also include a citation and full reference for the retraction notice.

Thank you for this review. The list is correct.

Reviewer #1: 1. Introduction: Paragraph 2. I recommend that the authors rephrase the second sentence to say "is rapidly inducible (~3-5 days) when carbohydrates are limited and primarily derived from non-starchy vegetables (20-50 g/day)." because one key component and nutrition recommendation for the Keto diet is low carbohydrate intake.

Thank you for your review. The authors agree and the sentence has been changed to the suggested version.

2. Methods: Is the last sentence of the first paragraph needed- “Consent to participate was obtained from each participant as written signatures.” It is common practice that enrolled participants in a clinical trial sign a consent form that he/she agrees to participate in the research study.

Thank you spotting this. The last sentence has been removed in agreement with your suggestion.

3. Methods. First sentence under the participants section- Please correct the spelling of enrollment.

Thank you for catching this oversight. This has been changed.

4. Methods: Last paragraph. The authors should remove the last paragraph. The paragraph discusses common practices to deidentify participants. This information should be encompassed in the IRB approval, and the information is not important for the overall methodology of the research study.

We thank the reviewer for their critique, however, the last paragraph of the version uploaded is a statistical analysis paragraph. The authors would like to remove any unnecessary paragraphs, but we cannot locate any paragraph about deidentifying methodology.

5. Methods: The following sentences under Blood Chemistry should be removed. This information is not needed, and removing the sentences will decrease the wordiness of the manuscript. 1. The 3rd and 4th sentences regarding the number of times the tubes were inverted. 4. The sentence regarding aliquoting plasma. 5. The last sentence about lancing and collecting a second blood droplet.

Thank you for your review. The authors agree and have removed the suggested sentences.

6. Results: Please report height in meters in Table 1 and Supplemental Tables 1-2.

Thank you for your review. Height has been added.

7. Results: The authors termed women who reported change in menses as “responders” and those who reported no change as “non-responders.” I suggest changing the terms responder and non-responder to something else. This terminology makes it seem that women who had no change in menses did not respond to the survey, and those who had change in menses responded to the survey. I suggest changing the term non-responders and responders to something else and re-titling Supplemental Table 1 to “Predictive Baseline Characteristics Between Reported Change in Menses vs. No Change in Menses.”

Thank you for your review. The authors agree and this has been changed.

8. Results: Please edit the sentence “The women who were randomized to the KD+PL diet attained clinically significant weight-loss at day 14, whereas by day 28 on the KD+KS and the LFD.” To “The women who were randomized to the KD+PL diet attained clinically significant weight-loss at day 14, whereas by day 28 weight loss was clinically significant for women on the KD+KS and the LFD.”

Thank you for your review. This sentence has been changed.

9. Results: Please define what clinically significant weight loss means.

Thank you for this review. The definition of clinically significant weight loss has been added.

10. Abstract: The women in this study are defined as overweight, yet your average BMI is >30. Please change this to overweight and obese.

Thank you for this review. The authors agree with the suggestion, and this has been added.

11. The full data set is not attached with this manuscript submission. I only see the attached tables with means and SDs.

Our data set has been made available here <https://doi.org/10.5061/dryad.sn02v6x9q> . This has been added to the manuscript.

Reviewer #2: Dear authors
It is a very good and valuable work, but if the following points are included in, it will be a very good paper.

We thank you for your comment and look forward to reading your review.
-The title doesn't match with the text. Because in your title, there is no mention of ketone supplementation and compared with a low fat diet (LFD).

Thank you for your review. The authors agree and LFD has been added to the title.

- The necessity and objectives of the research are not well stated at the end of the introduction, so there should be more discussion about supplementation and why it is used during menstruation.

Thank you for your review. Although this manuscript speaks specifically to the female participants of this study, the participants were extracted from a larger study that included both men and women. This exploratory analysis of all female participants was only derived when the notable change in menses across the Ketogenic Diet groups. The ketone salt supplementation was not included for any specific female hormonal purposes, but rather the metabolic changes in both men and women on a Ketogenic Diet and a Ketogenic Diet plus Ketone Salt supplement. The primary aim of the large study was to investigate the metabolic impacts on a hypocaloric ketogenic diet with and without ketones salts. Later, a hypocaloric low-fat diet arm was added for a control. Previous publications have reported the primary findings of the study [1, 2, 3, 4, 5]. However, the authors agree that further research is needed to specifically determine the effects of added supplementation on the female menstrual cycles.

- In the methods section, the method of prescribing and controlling diets must be described in more detail. How is ketosis status controlled?

Thank you for your inquiry. The authors have added further detail in regard to the controlled dietary composition of each group and have also included in the blood chemistry section that daily ketone and glucose measurements were taken to ensure participants were following the diet.

-The composition of macronutrients and total energy in each diet plan better to be mentioned and compared in a table.

Thank you for your comment. This has been added into the supplemental file.

-Which reference was used for the Menses Survey? Is it valid? Please give a reference.
We thank you for your review.

Given the exploratory approach of the initial study, our methods were intended to capture broader changes in both men and women, rather than focusing solely on women. Additionally, we utilized an instrument to record menstrual cycles that had not undergone prior validation or reliability testing. The authors agree that a validated survey would have been more applicable and plan to utilize menses tracking applications and validated surveys in our future work. This limitation was added in the original manuscript.

-It is better to be mentioned the Menses stages by the participants at the time of evaluation

Thank you for your review. Although this manuscript speaks specifically to the female participants of this study, the participants were extracted from a larger study that included both men and women. This exploratory analysis of all female participants was only derived when the notable change in menses across the Ketogenic Diet groups. We were not able to collect the phase at which the participants were in their cycle. Also, 7 of our participants hadn’t had a menstrual cycle in over a year, thus we would have been unable to determine their cycle. This limitation was added into the original manuscript.
