# Supplementary material for: Self-reported menses physiology is positively modulated by a well-formulated, energy-controlled ketogenic diet vs. low fat diet in women of reproductive age with overweight/obesity
Source: PLoS One. 2024 Aug 16;19(8):e0293670. doi: 10.1371/journal.pone.0293670 (PMC11329152; doi:10.1371/journal.pone.0293670)
Supplement: Attachment — Submitted filename: Response to Reveiwers 10092023.docx [file pone.0293670.s007.docx]

Dear Editor and Reviewers,

Thank you for your expertise and critique of our manuscript. We found your suggestions and requests extremely helpful. We have provided our answers and changes to each comment below in red.

Reviewer Comments:

Reviewer #1: The author addressed all my suggested revisions. I believe that this manuscript is ready for acceptance. My only reason to suggest minor revisions is because the link to the dataset did not work in my browser. It is the journal policy that the reviewers receive access to the dataset, and without this, I cannot suggest acceptance.

Thank you for this comment. The current link within the manuscript shows a link that will only be available when the manuscript is published. The temporary link for pre-publishing can be found here if you would like to see the data. <https://datadryad.org/stash/share/yPqBRNfIYlPetQpUKlTzqbGkJ4pz0oSOCf7zGRJ8Ksc>

Reviewer #2: Dear author
Thanks for complete correction of comments. But, need to two minor edit in the results section.

1. Is the table st3 for baseline measurements? If the answer is yes, it should be mentioned in the title of the table.

Thank you for your comment. The authors agree and this has been added.

2. I did not see your addressed Table st4 for the blood parameters section in the main text. Please give the address of all tables and figures in its own section in the main text.

Thank you for your review. ST4 was added in the Physiological Outcomes in the previous submission, but is again referenced in the blood parameter section. We hope this will suffice.
