# Supplementary material for: Self-reported menses physiology is positively modulated by a well-formulated, energy-controlled ketogenic diet vs. low fat diet in women of reproductive age with overweight/obesity
Source: PLoS One. 2024 Aug 16;19(8):e0293670. doi: 10.1371/journal.pone.0293670 (PMC11329152; doi:10.1371/journal.pone.0293670)
Supplement: Attachment — Submitted filename: Response to Reviewer 12072023.docx [file pone.0293670.s008.docx]

Dear Editor and Reviewers,

Thank you for your expertise and critique of our manuscript. We found your suggestions and requests extremely helpful. We have provided our answers and changes to each comment below in blue.

Major revisions:
In general, since the sample sizes are small and the distribution of the data cannot be shown to be normal, non-parametric tests should be used for comparisons among/between the groups. An underlying assumption of the t-test and ANOVA is normally distributed data.

We express our gratitude for your insightful review of our study. In our preliminary analysis, we meticulously considered the characteristics of our continuous data and adhered to the specific assumptions associated with parametric tests. Despite encountering small sample sizes, we observed that certain variables exhibited distributions that approximated normality.

For those variables demonstrating a reasonable approximation to normality, we opted for t-tests and ANOVA to capitalize on the advantages offered by these parametric tests, including enhanced statistical power. These tests, being sensitive to group differences, provided meaningful insights into the mean distinctions among various conditions. Nevertheless, we acknowledge the inherent heterogeneity in our some of our data and the constraints associated with small sample sizes.

It is worth noting that our decision to employ ANOVA was influenced by its robustness in controlling Type I errors, particularly in samples with less than 30 observations and involving three or more experimental conditions. The rationale for this choice aligns with findings discussed in the paper by Blanca et al. (2017), titled "Non-normal data: Is ANOVA still a valid option?" (Psicothema, 29(4), 552–557, https://doi.org/10.7334/psicothema2016.383).

Furthermore, ANOVA demonstrates low sensitivity to moderate departures from normality. Various simulation studies, such as those conducted by Glass et al. (1972), Harwell et al. (1992), and Lix et al. (1996), utilizing diverse non-normal distributions, have consistently shown that violating the assumption of normality does not significantly impact the false positive rate associated with ANOVA. These studies contribute to the confidence in the robustness of ANOVA even in the face of departures from normality.

We appreciate your attention to these methodological considerations, and we remain committed to providing a comprehensive and rigorous analysis of our data.

1- Statistics section: Clarify how the within-diets were quantified using Kruskal-Wallis tests since this test is for independent samples.

 Certainly, the Kruskal-Wallis tests were employed to quantify changes within diets and to compare differences between diets in the context of the menses survey responses. The Kruskal-Wallis test is a non-parametric test suitable for comparing more than two independent groups, making it an appropriate choice for analyzing the categorical responses obtained from the menses survey within and between the experimental diets.

In this study, the responses from the menses survey were categorized into four distinct groups: "no change," "change in frequency," "change in intensity," and "regained period after >1 year." These categories were treated as ordinal data, and the Kruskal-Wallis tests were utilized to assess if there were statistically significant differences in the distribution of these responses within each diet and between the diets at different time points (days 14, 28, and 42). The Kruskal-Wallis test is particularly robust for situations where assumptions of normality and homogeneity of variance may not be met, which is crucial when dealing with categorical data that may not follow a normal distribution. By employing this non-parametric test, the study aimed to identify if there were significant differences in menses-related changes within each diet group and whether these changes were significantly different between the ketogenic diets (KD+KS, KD+PL) and the low-fat diet (LFD).

The outcomes of these Kruskal-Wallis tests were then used to draw conclusions about the impact of the dietary interventions on self-reported menses changes. Specifically, the study found significant differences in menses frequency and/or intensity within the KD+KS and KD+PL groups compared to the LFD group at different time points. This information contributes to the understanding of how the interventions influenced subjective perceptions of menstrual events and supports the study's conclusions regarding the potential effects of the different diets on menstrual patterns.

2- Table 1: The sample sizes in the three groups are small; therefore, the Kruskal-Wallis test, a nonparametric test, would be more powerful when comparing the central tendencies of these baseline characteristics in the three groups.

Thank you for your insightful comment regarding the sample sizes in our study. We acknowledge the concern about small sample sizes within the three groups, and we appreciate the suggestion to consider the Kruskal-Wallis test for comparing central tendencies of baseline characteristics. We understand the importance of statistical power, especially in the context of nonparametric tests like the Kruskal-Wallis test. While our sample sizes are relatively small, it is essential to note that our study involves a specific population with unique dietary interventions and extensive assessments. Despite the challenges in recruiting larger cohorts, we have implemented rigorous statistical methods to draw meaningful conclusions from the available data.

To address the concern, we have conducted post hoc power analyses to assess the adequacy of our sample sizes for detecting significant differences. Additionally, we have included effect size estimates to provide a clearer picture of the practical significance of observed effects. We believe that the utilization of nonparametric tests, such as the Kruskal-Wallis test, is appropriate given the nature of our data and the potential deviation from normality.
3- If the interaction effect is significant, provide an interpretation of the results, but do not test main effects because the tests for main effects are uninteresting in light of significant interactions. If interaction effects are non-significant, drop the interaction effects from the model and test the main effects. Determining which results to present when testing interactions is often a multi-step process.

Thank you for your comment. The text has been corrected to the provided advise.

4- Table 2: An underlying assumption of the Kruskal-Wallis test is independent samples; therefore, it is not valid for comparing the data in Table 2.

Thank you for your comment regarding the Kruskal-Wallis test and its underlying assumption of independent samples. We appreciate the opportunity to address this concern. It is important to note that while the Kruskal-Wallis test assumes independent samples within each group, it is designed to handle situations where the assumption of normality or homogeneity of variances may be violated. In our study, the data in Table 2, particularly the responses from the menses survey, are categorical in nature, and the Kruskal-Wallis test is employed to assess differences among multiple independent groups.

The nature of our study design involves distinct experimental groups, and participants within each group are considered independent for the purpose of statistical analysis. The Kruskal-Wallis test is a non-parametric alternative suitable for comparing groups with small sample sizes and non-normally distributed data, making it an appropriate choice for our specific context. We have ensured that the assumptions of the Kruskal-Wallis test are met to the best extent possible within the constraints of our study. If there are specific concerns or alternative analyses you would recommend, we would be more than willing to consider and address them in our manuscript. We appreciate your diligence in reviewing our work and welcome any further guidance you may provide.

Minor revisions:
1- Abstract: Identify the statistical testing methods from which p-values were estimated.

Thank you for your comment. This has been added.

2- Menses section: A) In addition to the frequencies provide the corresponding percentages.
Thank you for your comment. This has been added.

B) Due to small sample sizes, compare BMI and serum MCP-1 in the two groups using non-parametric tests such as Wilcoxon rank sum tests.
This table has been added. Thank you for your comment.

3- Indicate the date range subjects participated in the study.
Thank you for your comment. This has been added.

4- State and justify the study’s target sample size with a pre-study statistical power calculation. The power calculation should include: (1) the estimated outcomes in each group; (2) the α (type I) error level; (3) the statistical power (or the β (type II) error level); (4) the target sample size, (5) the statistical testing method and (6) for continuous outcomes, the standard deviation of the measurements.
Our study, initially designed as an exploratory investigation, emerged from unexpected observations in the larger context of a study that included both men and women. The decision to focus specifically on women's responses was driven by the surprising findings related to menstrual cycle changes reported by female participants following ketogenic dietary interventions.

Given the exploratory nature of this inquiry and the unforeseen avenue it took, we acknowledge that a pre-study statistical power calculation was not conducted. The unexpected nature of the observed effects, particularly in the context of menstrual cycle changes, prompted this focused investigation on women. Our aim was to provide an in-depth exploration and analysis of the reported outcomes rather than adhering to a pre-determined sample size. We recognize the importance of statistical power calculations in ensuring the robustness of study designs. In the context of our unexpected findings, we addressed this limitation transparently in our manuscript.

5- To assist in the review process, add line numbering to the document and double space the lines.

We have added numbers. We hope this assists the reviewer!
